# Hearing disorders - findings on computed tomography and magnetic resonance imaging: pictorial essay

**DOI:** 10.1590/0100-3984.2016.0213

**Published:** 2019

**Authors:** Tiago Medina Salata, Bruno Niemeyer de Freitas Ribeiro, Bernardo Carvalho Muniz, Lívia de Oliveira Antunes, Heraldo Belmont Rosas, Edson Marchiori

**Affiliations:** 1 Hospital Casa de Portugal / Clínica 3D Diagnose, Rio de Janeiro, RJ, Brazil.; 2 Instituto Estadual do Cérebro Paulo Niemeyer, Rio de Janeiro, RJ, Brazil.; 3 Universidade Federal do Rio de Janeiro (UFRJ), Rio de Janeiro, RJ, Brazil.

**Keywords:** Hearing loss, Head and neck neoplasms, Neuroimaging, Tomography, X-ray computed, Magnetic resonance imaging, Perda auditiva, Neoplasias de cabeça e pescoço, Neuroimagem, Tomografia computadorizada, Ressonância magnética

## Abstract

Hearing disorders are usually unilateral and are more common in women. They can be congenital or acquired, and hearing loss is categorized as sensorineural, conductive, or mixed. The onset of hearing loss can be progressive or sudden, and it is a common reason for seeking medical attention. In this context, computed tomography and magnetic resonance imaging have assumed critical roles in the search for an etiological diagnosis and in guiding the therapeutic approach. In this pictorial essay, we illustrate the common causes of hearing loss, discussing the possible differential diagnoses and highlighting the most relevant imaging findings.

## Abstract (pt)

Distúrbios da audição são comumente unilaterais e mais comuns em mulheres, podendo ser de origem congênita ou adquirida, consequentes a distúrbios neurossensoriais, condutivos ou mistos, com evolução progressiva ou súbita, sendo uma motivação frequente de idas ao consultório médico. Dentro desse contexto, a tomografia computadorizada e a ressonância magnética assumiram fundamental importância na busca da definição etiológica e para guiar a conduta terapêutica. Neste ensaio iconográfico ilustraremos causas frequentes de déficit auditivo, demonstrando possíveis diagnósticos diferenciais e ressaltando os aspectos de imagem mais relevantes.

## INTRODUCTION

Hearing is of fundamental importance in the overall development of human beings, as it is considered one of the main senses. Any disorder that affects it, to any degree, has an adverse effect on functional status, quality of life, and cognitive function, as well as on emotional, behavioral, and social well-being.

During the diagnostic investigation, computed tomography (CT) and magnetic resonance imaging (MRI) of the temporal bone and of the brain play a fundamental role in a large portion of hearing disorders, helping to define the therapeutic approach.

There are many etiologies associated with hearing loss. In the initial evaluation, psychiatric and pharmacological causes should be excluded before any imaging evaluation is considered.

In this study, we will discuss the imaging findings according to the etiological classes, organized by cause: neoplastic, infectious/inflammatory, congenital, traumatic/post-surgical, and other.

## NEOPLASTIC CAUSES

### Schwannoma

**Figure 1:**
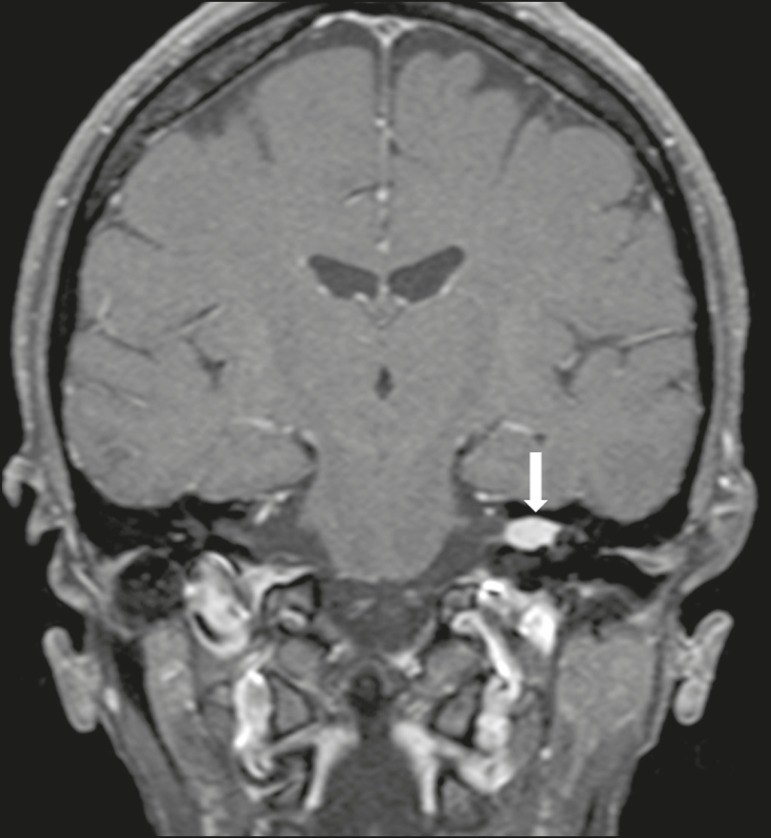
Intracanalicular schwannoma. Contrast-enhanced coronal T1-weighted MRI sequence showing an oval lesion with intense gadolinium enhancement, located in the internal auditory canal and resulting in its enlargement (arrow).

A schwannoma is the most common tumor of the cerebellopontine angle, usually presenting a homogeneous aspect when small. Its insinuation into and enlargement of the internal auditory canal is a suggestive but nonspecific aspect (Figure 1). The affected nerve is typically the eighth cranial nerve, or vestibulocochlear nerve, its involvement primarily manifesting as tinnitus and hearing loss.

### Paraganglioma

**Figure 2:**
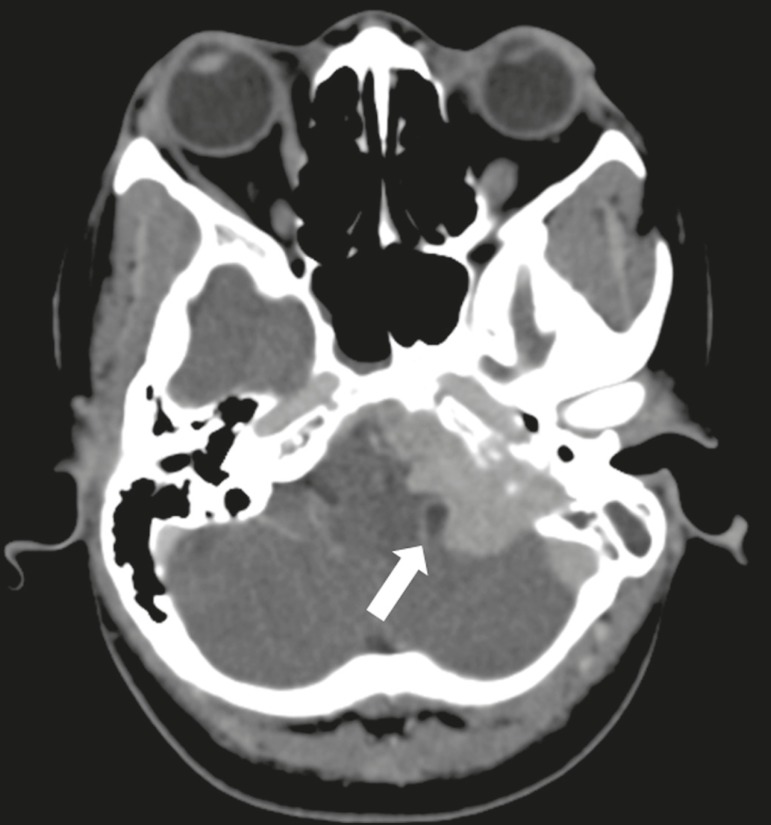
Paraganglioma. Axial CT scan, after intravenous administration of contrast medium, showing an expansile lesion with its epicenter in the jugular bulb, presenting intense contrast enhancement and resulting in erosion of the temporal bone (arrow).

Paragangliomas are tumors of the chemoreceptor system and are the main primary tumors of the jugular foramen. Although most paragangliomas are benign, they behave aggressively. On CT, they manifest as irregular bone destruction and significant contrast enhancement (Figure 2). On MRI, they present signals that are hypointense in T1-weighted sequences and hyperintense in T2-weighted sequences, with intense contrast enhancement. Larger lesions may contain flow voids.

### Endolymphatic sac tumor

**Figure 3:**
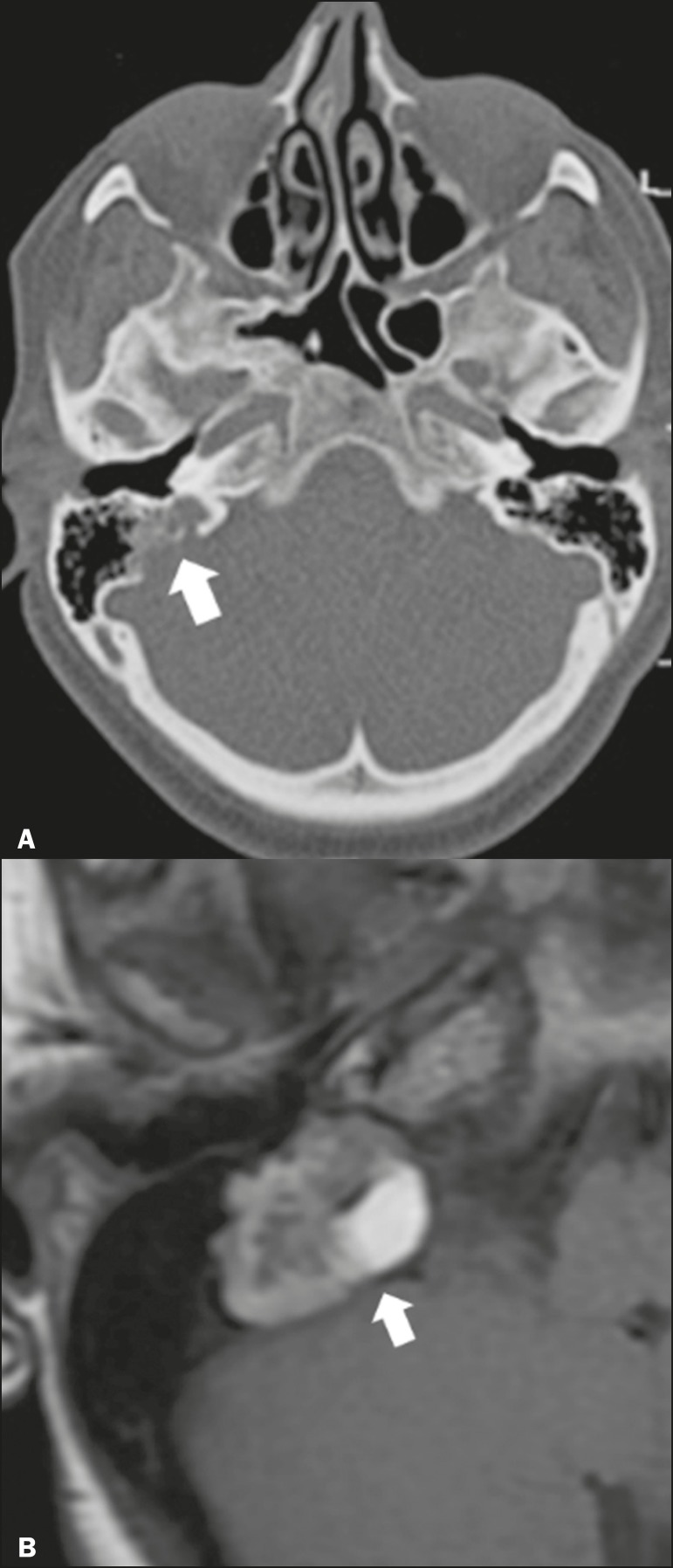
Endolymphatic sac tumor. **A:** Axial CT scan, with bone window settings, showing areas of bone erosion in the mastoid and labyrinthine segment of the temporal bone (arrow). **B:** Noncontrast T1-weighted MRI sequence showing a hyperintense signal, probably related to degradation products of hemoglobin and cholesterol (arrow).

Endolymphatic sac tumors are rare tumors of the posterior region of the petrous portion of the temporal bone that are slow-growing and occur sporadically in the majority of cases. Although not malignant, they are locally invasive. There is an association with von Hippel-Lindau disease in 15% of cases^(^1^)^. On CT, the bone destruction is either geographic or in a moth-eaten pattern, with a halo of peripheral calcification. On MRI, the signal is heterogeneous, with hyperintense foci within the lesion seen in T1-weighted sequences (Figure 3).

## INFECTIOUS/INFLAMMATORY CAUSES

### Cholesteatoma

**Figure 4:**
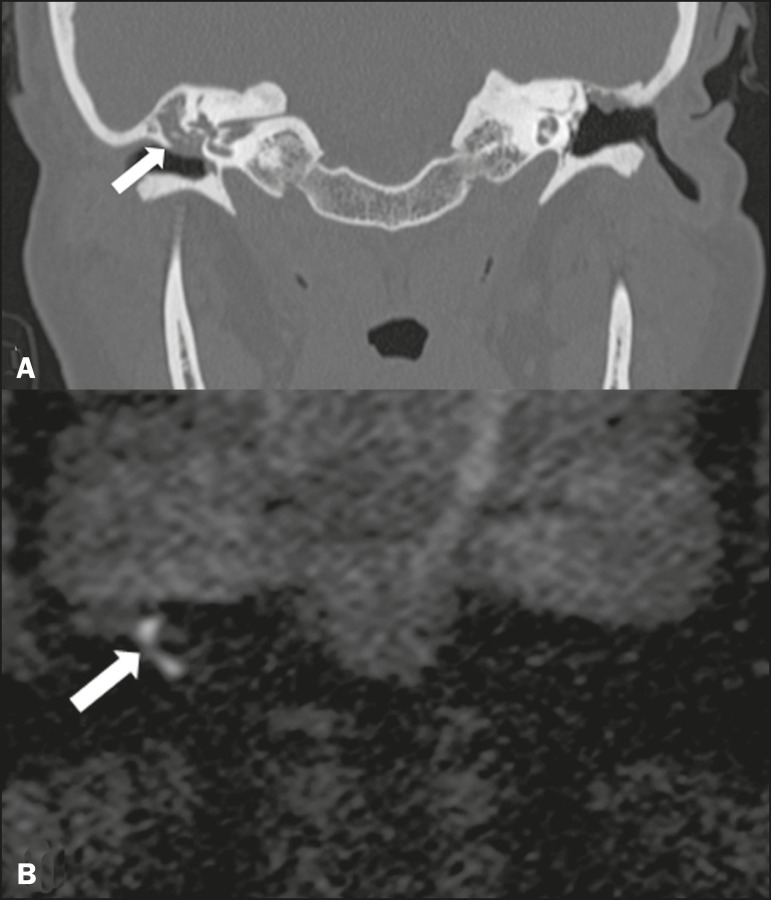
Cholesteatoma. **A:** Coronal CT scan, with bone window settings, showing hypodense material with soft-tissue density, affecting the tympanic cavity on the right and causing bone erosion (arrow). **B:** Coronal diffusionweighted MRI sequence showing restricted diffusion within the lesion identified on CT (arrow).

Cholesteatomas arise from the proliferation of keratinized squamous stratified epithelium, with pathological characteristics identical to those of an epidermoid cyst. They can be acquired or congenital, occurring either in the pars flaccida or pars tensa, those occurring in the former typically being acquired. On CT, they commonly appear as lesions with a soft-tissue density in Prussak's space, accompanied by erosion of the ossicular chain and lateral wall of the attic (epitympanic recess), with or without labyrinthine fistulae (Figure 4A). A functional diffusion-weighted MRI sequence without echo-planar imaging shows high sensitivity in the detection of lesions ≥ 2 mm, facilitating the distinction between cholesteatomas and inflammatory granulation tissue (Figure 4B).

### Otomastoiditis and osteomyelitis

**Figure 5:**
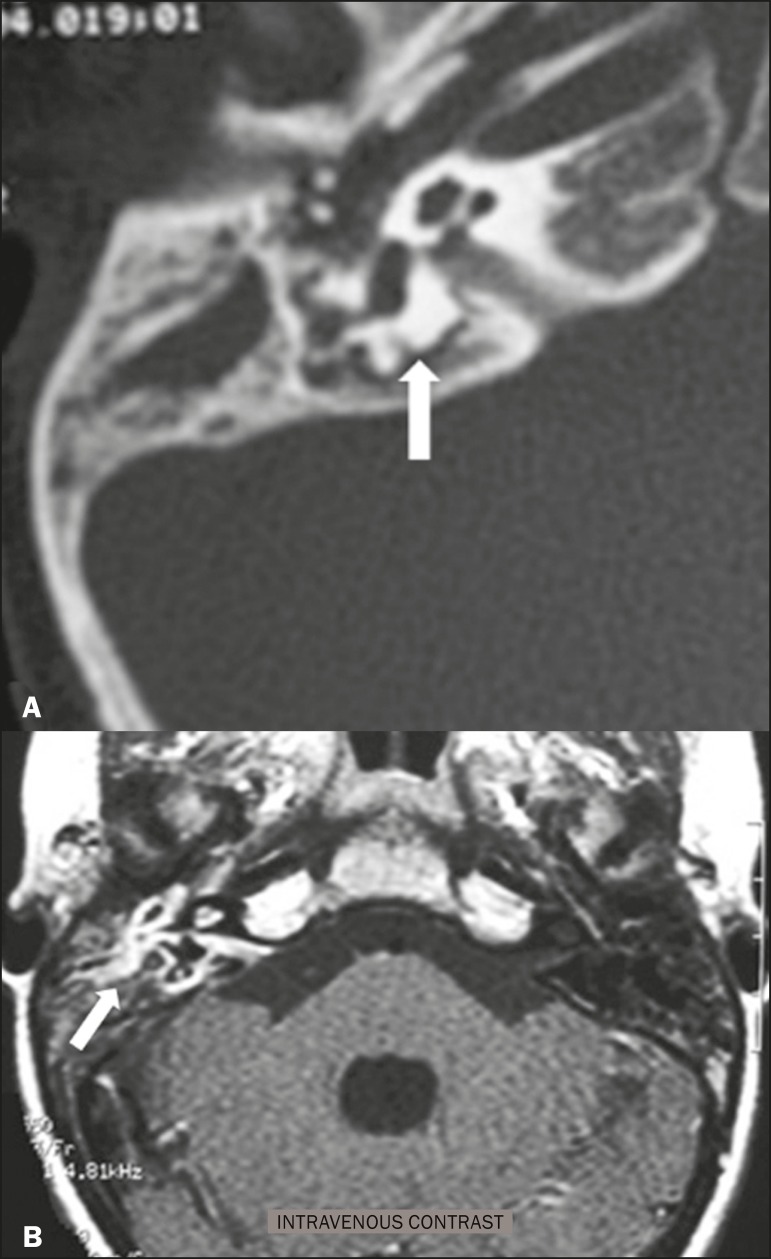
Otomastoiditis complicated by osteomyelitis. **A:** Axial CT scan, with bone window settings, showing right-sided otomastoiditis, surrounding a sequestrum (arrow). **B:** Contrast-enhanced axial T1-weighted MRI sequence showing intense contrast uptake (arrow).

Otomastoiditis and osteomyelitis consist of infection of the tympanic and mastoid cavities, mainly caused by bacteria. Immunocompromised patients present with risk factors for unusual infectious agents, in addition to more extensive and rapidly progressive impairment. On CT, uncomplicated otomastoiditis commonly manifests as hypodense material with no bone erosion. On MRI, no restricted diffusion is expected. When not properly treated, otomastoiditis can evolve to osteomyelitis or intracranial complications, including meningitis, abscesses, and venous thrombosis (Figure 5). The incidence of such complications has decreased substantially since antibiotics have come to be more widely used.

### Labyrinthitis ossificans

**Figure 6:**
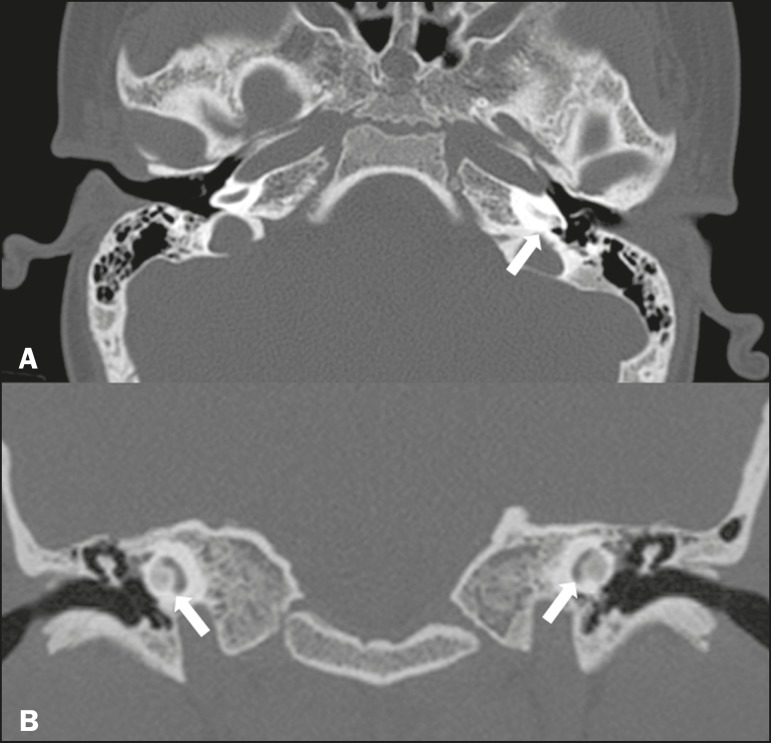
Labyrinthitis ossificans. **A:** Axial CT scan, with bone window settings, showing calcification involving the basal turn of the cochlea to the left (arrow). **B:** C oronal C T s can, w ith b one w indow s ettings, s howing c alcifications affecting both cochleae (arrows).

Labyrinthitis ossificans is the pathological ossification within the otic capsule, including the cochlea and the vestibule, caused by an inflammatory or destructive process. It can be related to infection (the most common cause), trauma, tumors, hemorrhage, otosclerosis, sickle cell anemia, or other factors^(^2^)^. MRI findings precede those of CT by some months, and are characterized by loss of the fluid signal within the membranous labyrinth (Figure 6A) and gadolinium enhancement in the earlier phases. On CT, foci with bone density within the labyrinthine canals in the inner ear can be seen (Figure 6B).

## CONGENITAL CAUSES

### Enlarged vestibular aqueduct

**Figure 7:**
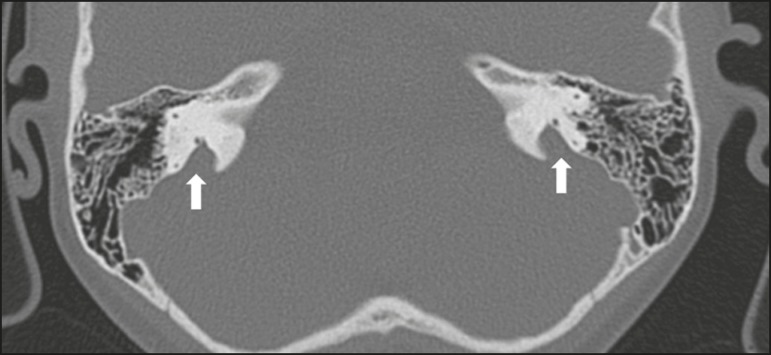
Enlarged vestibular aqueducts. Axial CT scan, with bone window settings, showing bilateral enlargement of the vestibular aqueduct (arrows).

An enlarged vestibular aqueduct is the most common congenital abnormality of the temporal bone and is associated with sensorineural hearing loss. It may be unilateral or bilateral, typically being identified at birth or in the first years of life. It occurs in isolation or in conjunction with syndromes. On CT, it is characterized by enlargement of the bone canal that extends from the vestibule to the posterior surface of the petrous portion of the temporal bone (Figure 7), vestibular aqueduct enlargement being defined as a diameter ≥ 1.5 mm at the midpoint or greater than the diameter of the adjacent posterior semicircular canal^(^3^,^^4^^)^.

### Other malformations of the outer, middle, and inner ear

**Figure 8:**
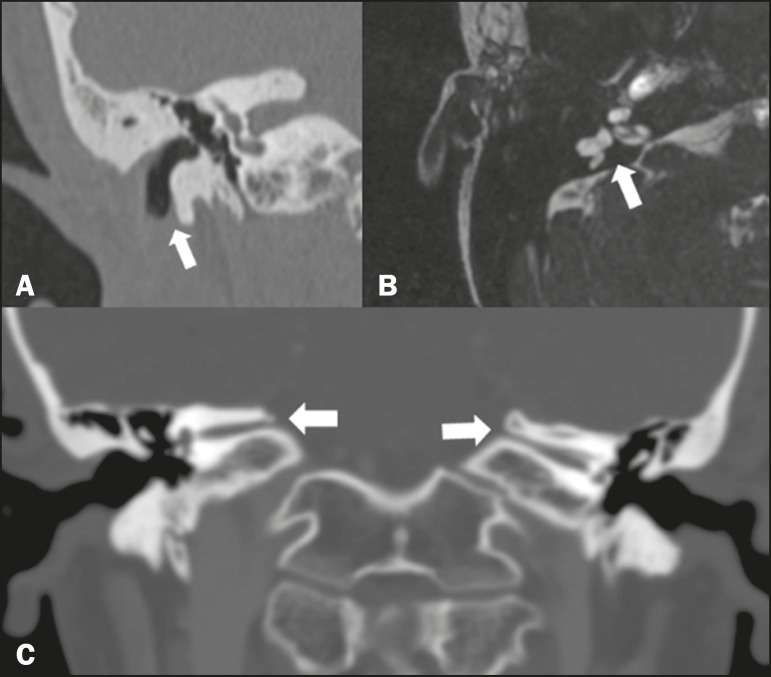
Congenital malformations. **A:** Coronal CT scan, with bone window settings, showing hypoplasia of the external auditory canal with stenosis of the membranous and bone portions, as well as vertical orientation of the canal (arrow). Note the thin atretic plate. **B:** Axial three-dimensional CISS MRI sequence showing the cochlea presenting fusion of the middle and apical turns, as well as an enlarged vestibule fused with the lateral semicircular canal. The superior and posterior semicircular canals are shortened and thickened (arrow). **C:** Coronal CT scan, with bone window settings, showing hypoplasia of the internal auditory canals (arrows).

Although CT is the method of choice in the evaluation of anomalies of the outer and middle ear, MRI is better in the evaluation of the membranous labyrinth and nerves within the internal auditory canal. Anomalies of the outer ear are commonly accompanied by middle ear alterations (Figure 8A), because both have a common origin. Congenital malformations of the outer and inner ear co-occur in 13-30% of cases^(^5^)^. Cochlear malformations are often accompanied by vestibular alterations (Figure 8B), and it is therefore more appropriate to classify them as vestibulocochlear malformations^(^6^)^. They can also be accompanied by anomalies of the internal auditory canal^(^6^)^ (Figure 8C).

## TRAUMATIC/POSTOPERATIVE CAUSES

### Fracture

**Figure 9:**
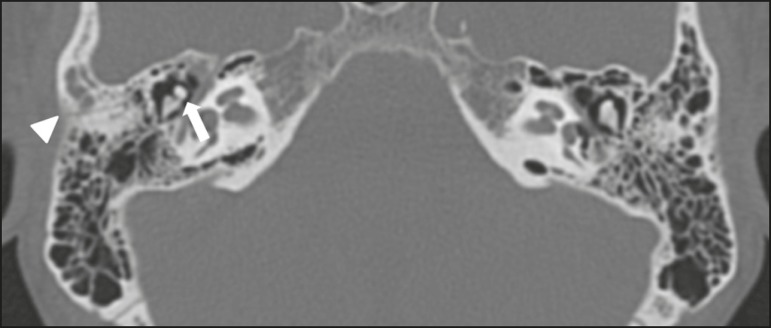
Fracture. Axial CT scan, with bone window settings, showing a longitudinal fracture line on the right (arrowhead), with involvement of the ossicular chain and subluxation of the incudomalleolar joint (arrow).

Most temporal bone fractures result from high-energy traumatic brain injury. The traditional classification indicates a relationship between the fracture line and the longest axis of the petrous portion of the temporal bone, these fractures thus being categorized as longitudinal, transverse, or mixed. Longitudinal fractures usually occur in temporoparietal trauma, affecting mainly the extralabyrinthine portion, the main complications being ossicular lesion and hemotympanum (Figure 9). Transverse fractures usually occur in fronto-occipital trauma and typically affect the translabyrinthine portion, with or without involvement of the facial nerve^(^7^,^^8^^)^.

### Postoperative displacement of a stapes prosthesis

**Figure 10:**
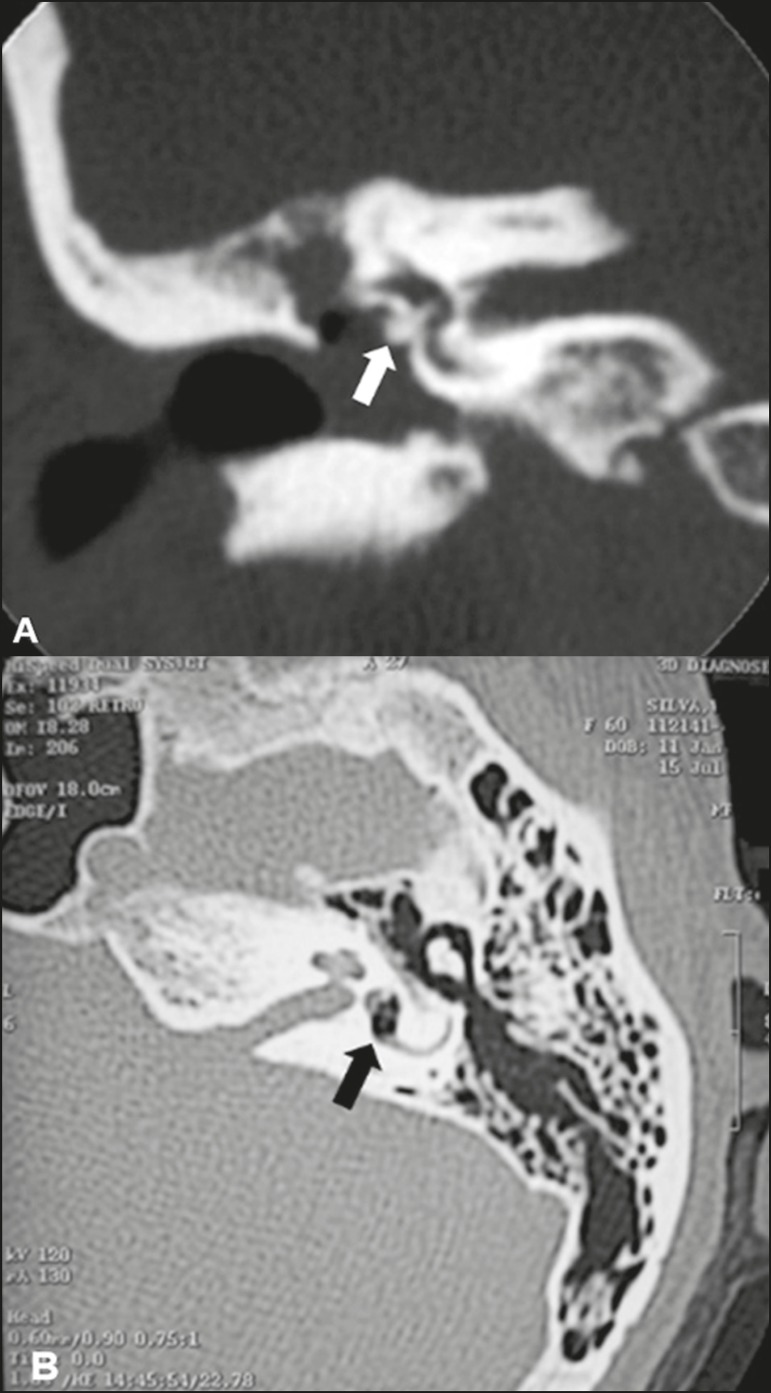
Displacement of a prosthesis. **A:** Coronal CT scan, with bone window settings, showing a stapes prosthesis insinuating itself through the oval window (arrow). **B:** Axial CT scan, with bone window settings, showing a small focus of gas inside the vestibule, indicative of a pneumolabyrinth (arrow).

Stapedectomy and reconstruction of the stapes is often used in order to restore conductive hearing loss in patients with otosclerosis or congenital anomalies. An ossicular prosthesis can malfunction weeks to years after surgery. CT helps identify the most common repairable causes of prosthesis failure, such as subluxation or luxation, prosthesis migration (often downward and posterior to the oval window), and displacement of the incudomalleolar joint^(^8^)^, as depicted in Figure 10.

## OTHER CAUSES

### Otosclerosis

**Figure 11:**
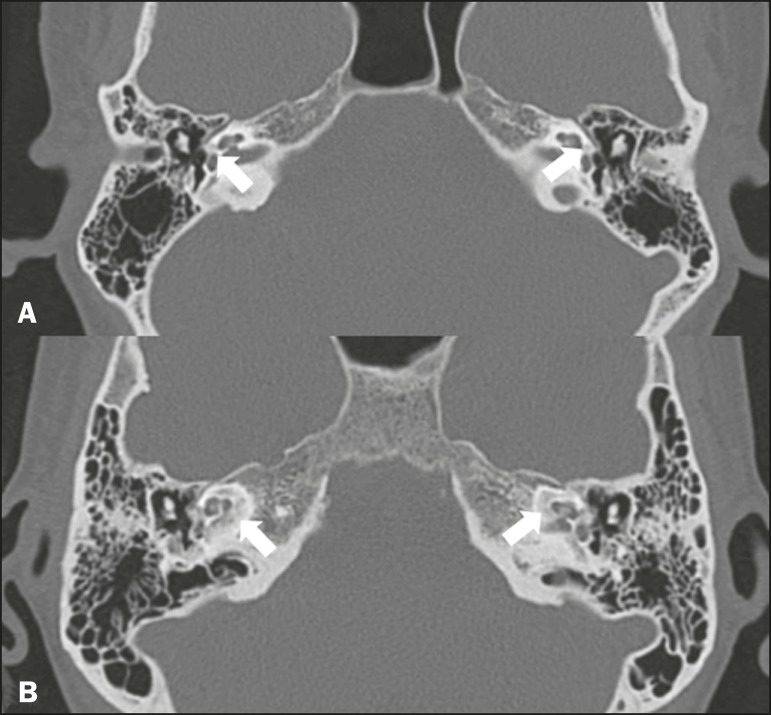
Otosclerosis. **A:** Axial CT scan, with bone window settings, showing a focus of demineralization in the region of the fissula ante fenestram bilaterally (arrows), characterizing fenestral otosclerosis. **B:** Axial CT scan, with bone window settings, showing pronounced bilateral pericochlear hypodensity (arrows), creating the "double-contour" sign, in a patient with cochlear otosclerosis.

Otosclerosis, also known as otospongiosis, is characterized by replacement of normal endochondral bone by vascular spongy bone in the otic capsule. It typically occurs between the ages of 10 and 39 years, is more prevalent in women, and is commonly bilateral (in 85% of cases). There are two types of otosclerosis-fenestral and retrofenestral/cochlear-the first being more related to conductive hearing loss and the second being related to sensorineural or mixed hearing loss^(^9^)^. In the fenestral type, noncontrast CT is the modality of choice and shows involvement of the lateral wall of the inner ear, mainly along the oval and round windows, the fissula ante fenestram being the region that is most affected (Figure 11). In the retrofenestral/cochlear type, the involvement is around the cochlea, and the fenestral type is typically also present.

### Paget's disease

**Figure 12:**
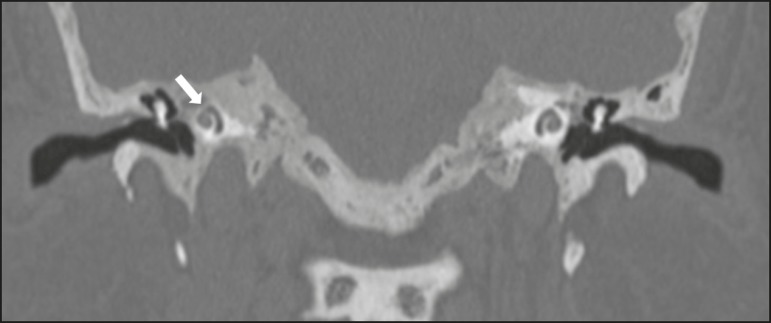
Paget's disease. Coronal CT scan, with bone window settings, showing intense pericochlear demineralization on the right (arrows).

Paget's disease is a focal disorder of bone remodeling, resulting in a disorganized, thickened bone structure with reduced mineral density. It is relatively common in the elderly population, and there is involvement of the temporal bone in 65-70% of the cases^(^10^)^. Deafness is the most common sensory deficit, occurring in 30-50% of patients^(^10^)^. On CT, Paget's disease manifests as cortical thickening and coarse trabeculation, with bone expansion and irregularities (Figure 12). On T1-weighted MRI sequences, the cortex is thickened and striated. Foci of osteolysis show hypointense signals in T1-weighted sequences and hyperintense signals in T2-weighted sequences^(^10^)^.

## CONCLUSION

CT and MRI are useful tools in the evaluation of patients with hearing loss and may add information essential to the diagnosis, treatment planning, and follow-up. The radiologist must be attentive to their indications, in order to be able to contribute to the clinical decision-making process.
